# Preliminary Report on Computed Tomography Radiomics Features as Biomarkers to Immunotherapy Selection in Lung Adenocarcinoma Patients

**DOI:** 10.3390/cancers13163992

**Published:** 2021-08-07

**Authors:** Vincenza Granata, Roberta Fusco, Matilde Costa, Carmine Picone, Diletta Cozzi, Chiara Moroni, Giorgia Viola La Casella, Agnese Montanino, Riccardo Monti, Francesca Mazzoni, Roberta Grassi, Valeria Grazia Malagnino, Salvatore Cappabianca, Roberto Grassi, Vittorio Miele, Antonella Petrillo

**Affiliations:** 1 Division of Radiology, Istituto Nazionale Tumori IRCCS Fondazione Pascale—IRCCS di Napoli, I-80131 Naples, Italy; v.granata@istitutotumori.na.it (V.G.); c.picone@istitutotumori.na.it (C.P.); a.petrillo@istitutotumori.na.it (A.P.); 2 Medical Oncology Division, Igea SpA, I-80013 Naples, Italy; 3 R & D Lab. of Tecnologie Avanzate TA Srl, Science and Technology Park, I-10153 Udine, Italy; matilde.costa@tecnologieavanzate.com; 4 Division of Radiodiagnostic, Azienda Ospedaliero-Universitaria Careggi, I-50134 Firenze, Italy; cozzid@aou-careggi.toscana.it (D.C.); moronic@aou-careggi.toscana.it (C.M.); vmiele@sirm.org (V.M.); 5 Italian Society of Medical and Interventional Radiology (SIRM), SIRM Foundation, I-20122 Milan, Italy; roberta.grassi@policliniconapoli.it (R.G.); roberto.grassi@unicampania.it (R.G.); 6 Division of Radiodiagnostic, Università degli Studi della Campania Luigi Vanvitelli, I-80128 Naples, Italy; giorgialacasella@glose.it (G.V.L.C.); riccardo.monti@studenti.unicampania.it (R.M.); salvatore.cappabianca@unicampania.it (S.C.); 7 Thoracic Medical Oncology, Istituto Nazionale Tumori IRCCS Fondazione Pascale—IRCCS di Napoli, I-80131 Naples, Italy; a.montanino@istitutotumori.na.it; 8 Division of Oncology, Azienda Ospedaliero-Universitaria Careggi, I-50134 Firenze, Italy; mazzonifr@aou-careggi.toscana.it; 9 Dipartimento Diagnosi e Terapia per Immagini, Radiologia Diagnostica, IRCCS Istituto Tumori G, Paolo II, I-70124 Bari, Italy; v.malagnino@oncologico.bari.it

**Keywords:** lung adenocarcinoma, radiomics, computed tomography, texture analysis, morphological analysis

## Abstract

**Simple Summary:**

The objective of the study was to assess the radiomics features obtained by computed tomography (CT) examination as biomarkers in order to select patients with lung adenocarcinoma who would benefit from immunotherapy. We demonstrated that specific radiomic features could be used to select patients with lung adenocarcinoma who would benefit from immunotherapy by predicting OS or PFS time.

**Abstract:**

Purpose: To assess the efficacy of radiomics features obtained by computed tomography (CT) examination as biomarkers in order to select patients with lung adenocarcinoma who would benefit from immunotherapy. Methods: Seventy-four patients (median age 63 years, range 42–86 years) with histologically confirmed lung cancer who underwent immunotherapy as first- or second-line therapy and who had baseline CT studies were enrolled in this approved retrospective study. As a control group, we selected 50 patients (median age 66 years, range 36–86 years) from 2005 to 2013 with histologically confirmed lung adenocarcinoma who underwent chemotherapy alone or in combination with targeted therapy. A total of 573 radiomic metrics were extracted: 14 features based on Hounsfield unit values specific for lung CT images; 66 first-order profile features based on intensity values; 43 second-order profile features based on lesion shape; 393 third-order profile features; and 57 features with higher-order profiles. Univariate and multivariate statistical analysis with pattern recognition approaches and the least absolute shrinkage and selection operator (LASSO) method were used to assess the capability of extracted radiomics features to predict overall survival (OS) and progression free survival (PFS) time. Results: A total of 38 patients (median age 61; range 41–78 years) with confirmed lung adenocarcinoma and subjected to immunotherapy satisfied inclusion criteria, and 50 patients in a control group were included in the analysis The shift in the center of mass of the lesion due to image intensity was significant both to predict OS in patients subjected to immunotherapy and to predict PFS in patients subjected to immunotherapy and in patients in the control group. With univariate analysis, low diagnostic accuracy was reached to stratify patients based on OS and PFS time. Regarding multivariate analysis, considering the robust (two morphological features, three textural features and three higher-order statistical metrics) application of the LASSO approach and all patients, a support vector machine reached the best results for stratifying patients based on OS (area under curve (AUC) of 0.89 and accuracy of 81.6%). Alternatively, considering the robust predictors (six textural features and one higher-order statistical metric) and application of the LASSO approach including all patients, a decision tree reached the best results for stratifying patients based on PFS time (AUC of 0.96 and accuracy of 94.7%). Conclusions: Specific radiomic features could be used to select patients with lung adenocarcinoma who would benefit from immunotherapy because a subset of imaging radiomic features useful to predict OS or PFS time were different between the control group and the immunotherapy group.

## 1. Introduction

For men, lung cancer is the leading cause of morbidity and mortality among oncological diseases; for women, on the other hand, it is third in incidence and second in mortality [1,2]. As first-line therapy in advanced non-small cell lung cancer (NSCLC), immunotherapy is used both as a single treatment and as a treatment in combination with chemotherapy.

The efficacy of immunotherapy in NSCLC and its pathophysiology has made evident over time the new cellular mechanisms associated with the response to treatment and to intrinsic resistance [3]. Moreover, bioinformatics analyses are becoming increasingly sophisticated, allowing the analysis and integration of complex clinical and biological data to further understand the biology of cancer, notably of lung carcinoma [3,4,5,6].

It is necessary to consider that even in the context of recent clinical scientific progress, the belief in clinical evidence of new robust biomarkers that predict response, resistance and/or toxicity to treatment in clinical care practice remains idealistic. Consequently, there is an urgent need to develop efficient biomarkers that can select patients who would benefit from immunotherapy, thereby providing the appropriate treatment and avoiding toxicity [3,6].

Radiomics is an emerging field, especially in the oncology field [7,8,9,10,11]. The radiomic approach has been used, in fact, in various research studies on pancreatic cancer [7], lung cancer [8,9], rectal cancer [10] and lymphoma [11].

The use of radiomics, as amply demonstrated by some studies, has been fundamental for predicting TNM and histological grade, response to therapy and survival in numerous oncological diseases [12,13,14].

It is inevitable to consider that by associating radiomic parameters with useful clinical and laboratory data, accurate and robust evidence-based clinical decision support systems (CDSS) could be established [15,16,17].

The radiogenomic approach (constituted by the combination of genomic data and radiomic metrics) [18,19] would allow the achievement of the most considerable level of precision medicine [20,21].

The primary endpoint of this study was to assess the efficacy of radiomics features obtained by computed tomography (CT) examination as biomarkers that could select patients with lung adenocarcinoma who would benefit from immunotherapy.

## 2. Materials and Methods

### 2.1. Patient Selection

The Local Ethics Committee of the National Cancer Institute of Naples, involving the National Cancer Institute of Naples Pascale Foundation and the Careggi University Hospital of Florence, with internal resolution no. 15 of 4 March 2019, approved a spontaneous multicenter retrospective study.

For the study, 74 patients (mean age 63 years, range 42–86 years) with histologically confirmed lung cancer who underwent immunotherapy (programmed cell death protein 1 (PD-1) and programmed death-ligand 1 (PD-L1) inhibitors) as first- or second-line therapy and a baseline CT study.

Because the study was performed in accordance with relevant guidelines and regulations, informed consent was not required by the Local Ethics Committee of the National Cancer Institute of Naples due to the retrospective nature of the study.

Inclusion and exclusion criteria are provided in Table 1.

As a control group, we selected 50 patients (median age 66 years, range 36–86 years) from 2005 to 2013 with histologically confirmed lung adenocarcinoma who underwent chemotherapy alone or combined with targeted therapy other than immunotherapy and who were subjected to a baseline CT study, including a CT venous phase protocol.

### 2.2. CT Protocol

Thanks to the use of 4 different scanners: General Electric Healthcare CT tomographs with 64 detectors (1 Optima 660 and 1 Discovery 750 HD, General Electric Healthcare, Milwaukee, Wisconsin, USA), 1 Philips CT scanner with 128 detectors (ICT SP 128 slice, Philips, Amsterdam, The Netherlands) and 1 Siemens CT scanner with 64 sections (Siemens Somatom Flash, Erlangen, Germany) it was possible to acquire computed tomography. Parameters of the CT scan data were already reported [22].

### 2.3. Radiological Assessment

Several radiologists with different levels of experience in reading and interpreting chest CT (low experience 5 years, average experience 5–15 years, and high experience ≥15 years) performed the radiological evaluations.

By selecting a single target lesion for each patient, the most visible lesion with the largest diameter was then analyzed.

Radiologists performed CT assessment using dedicated CT post-processing workstations and the HealthMyne^®^ software platform (www.healthmyne.com, accessed on 16 January 2020, HealthMyne, Madison, WI, USA). To reduce recall bias, all 3 readers maintained a gap of more than 2 weeks between the 2 sessions.

### 2.4. CT Post-Processing with Radiomic Precision Metrics (RPM™) Tool

**Figure 1:**
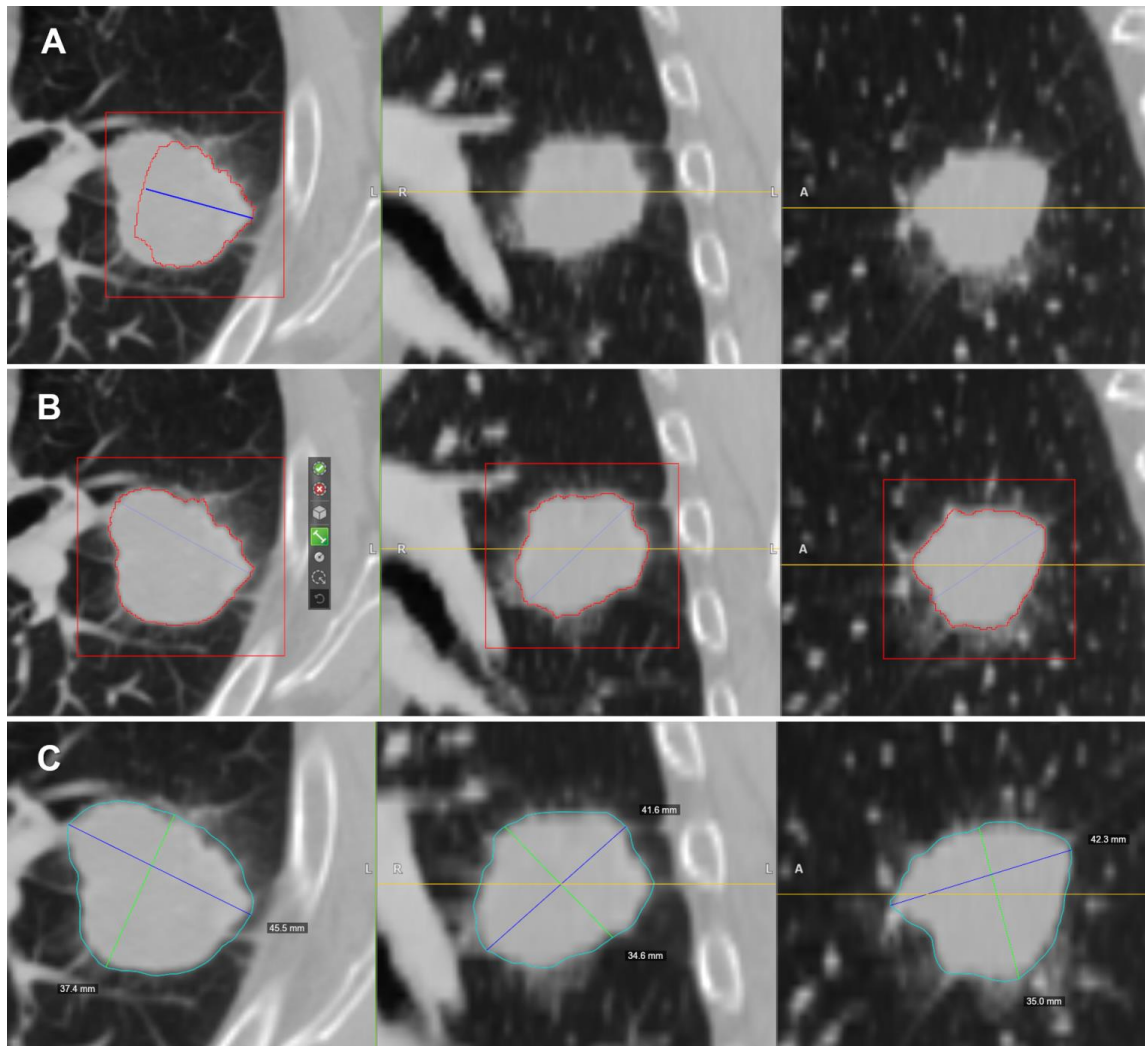
Semi-automatic lesion identification: (**A**) Manual ROI indication. In blue, it is possible to observe the axes that cross the lesion manually delineated by the radiologist on a plane of the MPR. The intensity of the lesion boundary (estimated) is represented with a red outline. (**B**) Additional axes can be dragged onto other orthogonal MPR views. From left to right, it is possible to observe the initial long axis outlined by the radiologist and the 2D contours on the axial, coronal and sagittal views of the lesion used as a starting point for the RPM ™ algorithms. (**C**) Resulting 3D contour of the lesion (in blue).

**Figure 2:**
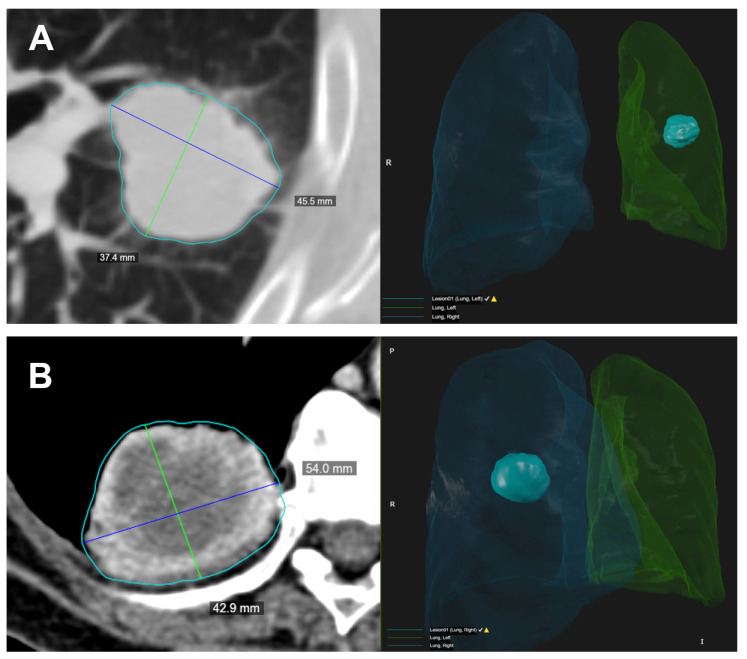
Two examples (**A**,**B**) of the semi-automated identification of the target lesion. On the left, the CT image with the lesion segmentation (light blue contour) and the longest diameters measured on the lesion volume. The blue lines represent the longest long axes and the green lines represent the longest short axes on the axial direction. On the right, it is possible to observe the 3D rendering of the lesion volume and its location inside the automatic lung segmentation.

In this study we used the HealthMyne^®^ platform for lesion segmentation and for radiomic features extraction from the delineated volumes of interest (VOIs). By means of the RPM™ algorithms, it was possible to semi-automatically recognize and segment the volume of the target lesions identified by the radiologist and automatically extract a wide range of quantitative data. The user initialized the lesion segmentation by drawing a long axis on a plane of the multiplanar reconstruction (MPR) (Figure 1A). A 2D segmentation updated in real-time with interactive feedback of the lesion boundary [23,24] and 2D segmentations on the other MPR planes were immediately proposed. When the contour on a MPR plane was unsatisfactory, the user could update the segmentation by either drawing long axes on the other MPR views or using a 2D brush tool (Figure 1B). When the segmentation was satisfactory, the user could confirm to initiate the 3D segmentation computation. Based on these initial user interactions, the RPM™ algorithms combined statistical sampling methods together with deep learning strategies in order to delineate the target volume and provide an automatic 3D segmentation (Figure 1C). The 3D segmentation occurred quickly (approximate time = 1–2 s), and could be reviewed by scrolling through slices on the MPR views. Interactive editing tools including 2D and 3D brushes could be used to reduce/enlarge or add details to the proposed volume segmentation. As the 3D segmentation was confirmed by the user, the measure of the long and short lesion axes was automatically determined by leveraging the volume delineation (Figure 2).

A total of 573 radiomic metrics were extracted from the delineated VOIs as previously reported in [24]: 14 features based on Hounsfield unit (HU) values specific for lung CT images; 66 first-order profile features based on intensity values (statistical distribution of image value); 43 second-order profile features based on lesion shape (geometric analysis of shape, volume, curvature and volumetric length); 393 third-order profile features, i.e., texture features, with IBSI-consistent implementation [25] of the grey-level co-occurrence matrix (GLCM), the grey-level distance zone matrix (GLDZM), the grey-level run length matrix (GLRLM), the grey-level size zone matrix (GLSZM), the neighboring grey-level dependence matrix (NGLDM), the neighboring grey-tone difference matrix (NGTDM) and the different features’ aggregation methods, as well as 57 features with higher-order profiles (statistical metrics after transformations and wavelet analysis).

### 2.5. Statistical Analysis

#### 2.5.1. Univariate Analysis

Overall survival (OS) was defined as the time between the date of first dose of therapy and the date of death or date of last clinical follow-up. Similarly, progression-free survival (PFS) was measured from the date of first dose of therapy to the time of tumor progression, recurrence, death or the time the patient was last known to be alive. The estimate of overall survival and progression-free survival was calculated with Kaplan—Meier analysis.

For each metric, median and range values were calculated.

The calculation of inter-observer variability between readers by intraclass correlation coefficient (ICC) and the evaluation of unstable features were performed.

Cox proportional hazard models were used for exploring univariate associations between OS and each stable imaging feature (identified as ICC value ≥0.8) and between PFS and each stable imaging feature (identified as ICC value ≥0.8). The evaluation between the survival rate and the variables was done using a technique called Cox regression analysis.

The risk measure provided for each variable was the risk ratio (RR): a RR of 1 means that the risk is the same for each participant; a RR >1 indicates higher risk; a RR <1 indicates lower risk.

A Wilcoxon–Mann–Whitney U test was performed to identify differences among imaging radiomic metrics of two groups (immunotherapy group and control group). A non-parametric Kruskal—Wallis test was performed to identify the significant features for stratifying the patients into two groups based on median cutoff of survival time (i.e., OS = 32 months and PFS = 10 months) corresponding to short (i.e., <median survival time) or long survival time (i.e., ≥median survival time).

Receiver operating characteristic (ROC) analysis was performed. The Youden index was used to individuate the optimal cutoff value for each feature and area under the ROC curve (AUC), sensitivity (SENS), specificity (SPEC), positive predictive value (PPV), negative predictive value (NPV) and accuracy (ACC) were obtained, considering the optimal cutoff value.

The statistical analyses were performed using the Statistics Toolbox of MATLAB R2007a (MathWorks, Natick, MA, USA).

#### 2.5.2. Multivariate Analysis

For multivariate analysis, we considered all stable significant features of univariate analyses as inputs for a classifier model. Pattern recognition methods (linear discrimination analysis (LDA), support vector machine (SVM), *k*-nearest neighbor (KNN), artificial neural network (ANN) and decision tree (DT)) were considered to assess the survival prediction ability [26]. The best model was chosen considering the highest area under the ROC curve and highest accuracy. Moreover, the analysis was made before and after a feature selection method: the robust features were selected by the least absolute shrinkage and selection operator (LASSO) method [27,28]. In the LASSO method, 10-fold cross-validation was used to select the optimal regularization parameter alpha, considering that the average of each patient’s mean square error was the smallest. With the optimal alpha, features with a nonzero coefficient in LASSO were reserved. Feature selection was carried out considering the λ value with the minimum mean square error (minMSE) [29,30].

A 10-k-fold cross validation approach was used to individuate the best classifier on the training set; therefore, median and 95% confidence interval values of AUC, accuracy, sensitivity, and specificity were calculated.

Multivariate analysis was performed using the statistics and Machine Learning Toolbox of MATLAB R2007a (MathWorks, Natick, MA, USA).

## 3. Results

Thirty-eight patients (median age 61; range 41–78 years) with confirmed lung adenocarcinoma and subjected to immunotherapy satisfied the inclusion criteria. We excluded: (a) 19 patients since the histological diagnosis was other than adenocarcinoma, (b) 17 patients since the baseline CT studies were not performed with contrast media.

### 3.1. Univariate Analysis Results

**Figure 3:**
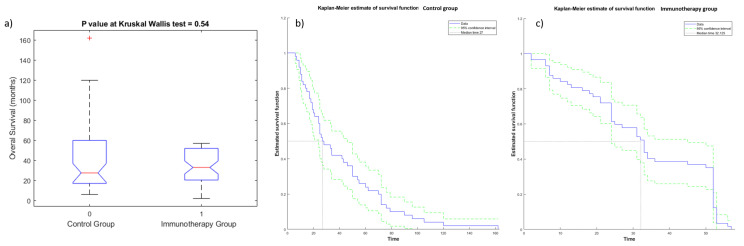
Boxplot (**a**) and Kaplan—Meier curve of OS for patients of control group (**b**) and of immunotherapy group (**c**).

**Figure 4:**
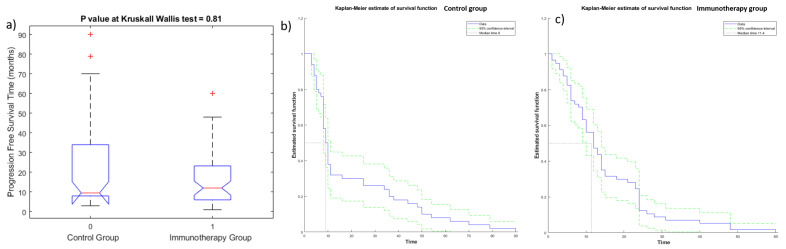
Boxplot (**a**) and Kaplan—Meier curve of PFS for patients of control group (**b**) and of immunotherapy group (**c**).

The Kruskal—Wallis test did not detect statistically significant differences in OS (Figure 3a) and PFS values (Figure 4a) among the two groups (immunotherapy group and control group), demonstrating the homogeneity among the two patient groups. Kaplan—Meier curves of OS and PFS are shown, respectively, in Figure 3b,c for the immunotherapy and control group and in Figure 4b,c for the immunotherapy and control group. The median value of OS for the immunotherapy group was equal to 32 months (range 2–72 months), while the median value of OS in the control group was 28 months (range 6–162 months). The median value of PFS for the immunotherapy group was equal to 12 months (range 1–60 months), while median value of PFS in the control group was 10 months (range 3–162 months).

Stable features (intraclass correlation coefficient value ≥ 0.8) were 121 among 573 calculated (see Appendix A for the description of each stable feature): 5 lung CT features, 26 morphological features, 1 feature based on intensity values, 76 texture features and 13 higher-order statistical features. The median value of intraclass correlation coefficients for stable features was 0.9 (range 0.85–0.96). Median size of lesions was 3 cm, with range of 1.0–12 cm. The size of the lesion did not affect the stable metrics (*p* value > 0.05 at the Wilcoxon–Mann–Whitney U test performed between the groups obtained by dividing patients with lesions < 3 cm and patients with lesions ≥ 3 cm).

Using Cox proportional hazard models, we found significant radiomic features to predict OS and PFS time in both groups (see Table 2 and Table 3): exclusively textual features including higher-order statistical metrics were significant in the Cox proportional hazard model. No metrics in the control group had a risk ratio > 1 to predict OS, while only one textural metric in immunotherapy group had a risk ratio > 1 to predict OS (Table 2): the grey-level nonuniformity as volume, with full merging by grey-level size zone matrix (GLSZM_IBSI_GL_NONUNIF_3D_HU GLSZM).

Several radiomic textural metrics in the immunotherapy group had a risk ratio >1 to predict PFS, while only one textural metric in the control group had a risk ratio >1 to predict PFS (Table 3): the NGLDM GL nonuniformity by slice, with merging by slice by neighboring grey-level dependence matrix (NGLDM_IBSI_GLNONUNIF_2DV_HU).

With regard to ROC analysis, we considered only the most important features. Table 4 reports the subset of significant features from Kruskal—Wallis tests for stratifying the patients into two groups based on median cutoff of OS time (short and long OS time). A total of 19 features were significant (3 morphological features, 1 feature based on intensity value, 12 textural metrics and 3 higher-order statistical metrics) to predict overall survival time. Among these 19, the best feature for stratifying the patients with short or long OS time was a higher-order statistical metric: the mean value of 2D Laplacian of Gaussian transformed voxels at 2.5 mm of smoothing (LOG_2D_MEAN_2_5MM_HU) with an AUC of 66.0%, a sensitivity of 69.0% and a specificity of 65.0%.

Table 5 reports the subset of significant features from Kruskal—Wallis tests for stratifying the patients into two groups based on median cutoff of PFS time (short and long PFS time). A total of 104 features (5 lung CT features, 23 morphological features, 1 feature based on intensity value, 64 textural features and 11 higher-order statistical metrics) were significant in predicting PFS time. Among these 104, the best feature for stratifying the patients based on PFS time was a textural feature: the average energy of gray-level co-occurrence matrix (GLCM_ENERGY) with an AUC of 70.0%, a sensitivity of 73.0% and a specificity of 64.0%.

The shift in the center of mass of the lesion due to image intensity (SHIFT_CENTER_OF_MASS_MM) was significant for predicting OS in patients subjected to immunotherapy and also for predicting PFS in both groups (patients subjected to immunotherapy and patients in the control group).

### 3.2. Multivariate Analysis Results

Regarding multivariate analyses, only the most useful results considering the purposes of this study are reported. Using all stable significant features, no tested classifier reached higher accuracy than a single radiomics feature for stratifying patients based on OS and PFS time (short or long survival time).

**Figure 5:**
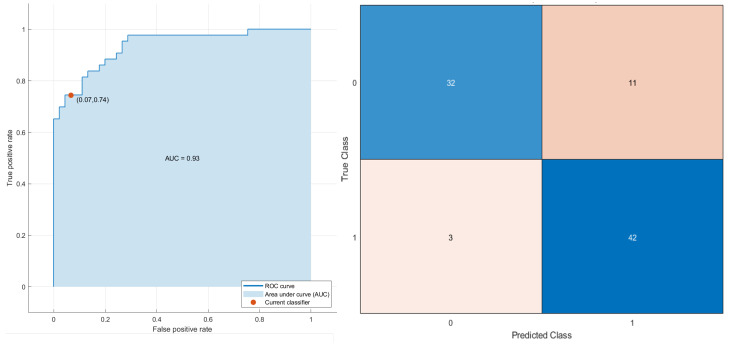
ROC curve and confusion matrix of SVM as the best classifier for stratifying the patients based on OS time (short and long time).

Considering the robust predictors by the LASSO approach and all patients, an SVM (Figure 5) reached the best results for stratifying patients based on OS time, with an AUC of 0.93 (0.85–0.96 95% confidence interval (CI)), an accuracy of 84.1% (80–86% 95% CI), a sensitivity of 74.4% (69–78% 95% CI) and a specificity of 93.3% (88–95% 95% CI). The robust predictors as input to the SVM totaled seven, including two morphological features, two textural features and three higher-order statistical metrics: greatest planar axis; volume fraction of the approximate enclosing ellipsoid occupied by the ROI (VOLUME_DENSITY_AEE); two features by GLCM cluster prominence for grey-leveled image from IBSI by slice (GLCM_IBSI_CLUSTERPROMINENCE_2DS_HU and complexity from averaging metrics by neighborhood gray-tone difference matrix (NGTDM_COMPLEXITY_2DF_HU)); median value of voxels under wavelet transforms with filters HHL (WAVELET_HHL_MEDIAN_HU); minimum value of voxels under wavelet transforms with filters (HHL WAVELET_HHL_MIN_HU); and the mean value of 2D Laplacian of Gaussian transformed voxels at 2.5 mm of smoothing (LOG_2D_MEAN_2_5MM_HU). The SVM classifier in the subset of patients treated with immunotherapy reached an AUC of 0.89, an accuracy of 81.6%, a sensitivity of 82.4% and a specificity of 81.0%.

**Figure 6:**
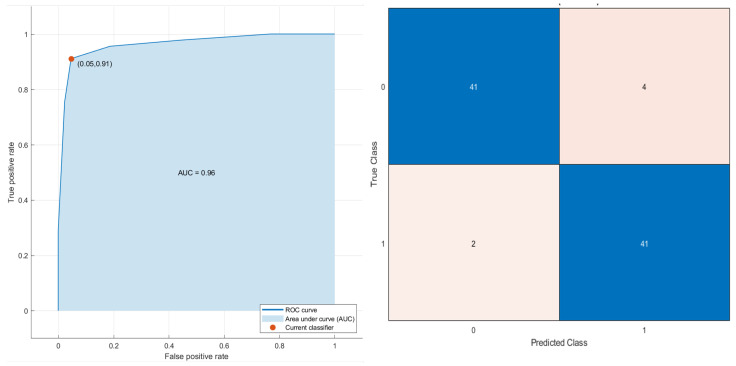
ROC curve and confusion matrix of decision tree as the best classifier for stratifying the patients based on PFS time (short and time).

Conversely, considering the robust predictors by the LASSO approach and all patients, a decision tree (Figure 6) reached the best results for stratifying patients based on PFS time with an AUC of 0.96 (0.895–1.0 95% confidence interval (CI)), an accuracy of 93.2% (88–96% 95% CI), a sensitivity of 91.1% (87–94% 95% CI) and a specificity of 5.3% (90–99% 95% CI). The robust predictors as inputs of SVM totaled seven (six textural features and one higher-order statistical metric):

Grey-level variance by neighborhood grey-level difference matrix (NGLDM_IBSI_DEP_VARIANCE_2DF_HU); average correlations of GLCM (GLCM_IBSI_CORRELLATION_2DS_HU); grey-level non-uniformity in three dimensions (GLDZM_IBSI_ZONE_DISTANCE_NONUNIFORMITY_3D_HU); entropy value in three dimensions by neighborhood grey-level difference matrix (NGLDM_IBSI_DEP_ENTROPY_3D_HU); high grey-level run emphasis in three dimensions by neighborhood grey-level difference matrix (NGLDM_IBSI_HIGH_DEP_LOW_GL_EMPH_3D_HU); strength value by slice with full merging by neighborhood grey-level difference matrix (NGTDM_STRENGTH_2DV_HU); the mean value of 2D Laplacian of Gaussian transformed voxels at 2.5 mm of smoothing (LOG_2D_MEAN_2_5MM_HU). The decision tree classifier in the subset of patients treated with immunotherapy reached an AUC of 0.96, an accuracy of 94.7%, a sensitivity of 87.5% and a specificity of 100.0%.

## 4. Discussion

Immunotherapy, capable of stimulating the cellular immune response against cancer, uses immune checkpoint blockade (ICI), a treatment paradigm in advanced cancer treatment.

The two main groups of agents used almost exclusively in tumors [31,32] are programmed cell death protein 1 (PD-1) and programmed death-ligand 1 (PD-L1) inhibitors; some authors analyzed the results of these ligands in small cell lung cancer [33,34,35]. Despite this, resistance to primary therapy does not allow all patients to benefit from the treatment regimen.

Considering this, it is therefore necessary to identify biomarkers that allow the appropriate selection of patients and the right stratification. Radiomics can therefore effectively support precision medicine decisions by identifying imaging biomarkers. Indeed, radiomics consists in the extraction of many quantitative characteristics through medical images [36]. This quantitative analysis, considering the heterogeneity of the macroscopic features based on the image [37], can identify the overall tumor.

The division into segments is not an immediate step in the whole of the radiomic process because the subsequent extraction of the characteristics is obtained from the segmented VOI. Lately, plot analysis has broadened its application to medical applications [37]. The quantification of grayscale patterns and pixel interrelationships that provide a measure of heterogeneity is what is called texture analysis.

In this study we evaluated 573 radiomic features; among them 121 were stable: 5 lung CT features, 26 morphological features, 1 feature based on intensity values, 76 texture features and 13 higher-order statistical features. Considering Cox proportional hazard models, textual features including higher-order statistic metrics were exclusively significant.

Considering Kruskal—Wallis tests, 19 radiomic features (3 morphological features, 1 feature based on intensity value, 12 textural metrics and 3 higher-order statistical metrics) were significant for predicting overall survival time. The best feature for stratifying the patients with short or long OS time was a higher-order statistical metric: the mean value of 2D Laplacian of Gaussian transformed voxels at 2.5 mm of smoothing (LOG_2D_MEAN_2_5MM_HU), with an AUC of 66.0%, a sensitivity of 69.0% and a specificity of 65.0%.

Considering Kruskal—Wallis tests, 108 radiomic features (5 lung CT features, 23 morphological features, 1 feature based on intensity value, 64 textural features and 11 higher-order statistical metrics) were significant for predicting PFS time. The best feature for stratifying the patients based on PFS time was a textural feature: GLCM ENERGY, with an AUC of 70.0%, a sensitivity of 73.0% and a specificity of 64.0%.

However, the subset of imaging radiomic features for predicting OS or PFS time was different in the control group and immunotherapy group; this demonstrated that specific radiomic features could be used to select patients with lung adenocarcinoma who would benefit from immunotherapy.

Exclusively, the shift in the center of mass of the lesion due to image intensity (SHIFT_CENTER_OF_MASS_MM) was significant both for predicting OS in patients subjected to immunotherapy and for predicting PFS in patients subjected to immunotherapy and in patients in the control group.

However, in univariate analysis, low diagnostic accuracy was reached for stratifying patients based on OS and PFS time.

A multivariate analysis using all stable significant features found that no tested classifier reached higher accuracy than a single radiomic feature for stratifying patients based on OS and PFS time (short or long survival time). Conversely, considering the robust predictors by the LASSO approach and all patients, an SVM reached the best results for stratifying patients based on OS. The SVM classifier in the subset of patients treated with immunotherapy reached an AUC of 0.89, an accuracy of 81.6%, a sensitivity of 82.4% and a specificity of 81.0%. However, considering the robust predictors of the LASSO approach and all patients, a decision tree reached the best results for stratifying patients based on PFS time. The decision tree classifier in the subset of patients treated with immunotherapy reached an AUC of 0.96, an accuracy of 94.7%, a sensitivity of 87.5% and a specificity of 100.0%.

The relationship between radiomics and immunotherapeutic response was demonstrated by numerous experts.

Radiomic signatures could be considered critically important inputs as a biomarkers for immune profiles and immune checkpoint inhibition response, according to a multi-center retrospective study on advanced cancers that considered all advanced cancers, including lung cancer [38].

Consequently, in a sample of 200 advanced NSCLC patients who received single anti-PD-1/PD-L1, Yang et al. assessed 1633 CT scans and 3414 blood samples, including serial radiomics, laboratory data and baseline clinical data, to build deep learning models useful for the selection and identification of responders and non-responders to immunotherapy. They found that a deep learning-based prediction model showed a good performance in distinguishing responders from non-responders to anti-PD-1/PD-L1 therapy [39].

In patients treated with the anti-PD-1 antibody, by combining PD-L1ES with a clinical model that was constructed using age, sex, smoking history and family history of malignant tumors, the reaction to immunotherapy could be anticipated in a manner more accurate than using PD-L1ES or the clinical model alone as predictors [40].

Accordingly, Tian et al. conducted analyses on PD-L1 expression in 939 consecutive stage IIIB–IV NSCLC patients with baseline CT images and found that deep learning on computed tomography images could predict a high expression of PD-L1 (PD-L1 ≥50%), with an AUC of 0.78.

The present study has several limitations: the small population size considered, the retrospective nature of the study and the awareness that CT images were collected by different centers and thus were usually obtained using different protocols. The radiomic model can be affected by these differences; radiomics data were not correlated and combined with clinical information.

## 5. Conclusions

With the contribution of medical images usually acquired in clinical practice, radiomics can be a useful support for precision medicine.

We demonstrated that specific radiomic features extracted by CT could be used to select patients with lung adenocarcinoma who would benefit from immunotherapy; in fact, the subset of radiomic features able to predict OS or PFS time was different in the control group and immunotherapy group.

## Figures and Tables

**Table 1:** Inclusion and exclusion criteria.

Inclusion Criteria	Exclusion Criteria
Lung adenocarcinoma histologically confirmed	Baseline CT study is not accessible
Lung nodule size > 10 mm	Tumor histology other than adenocarcinoma
Immunotherapy ((PD-1)/programmed death-ligand 1 (PD-L1) inhibitors) as first- or second-line therapy	
CT examination within 1 month of immunotherapy	
CT protocol included venous phase (70–90 s post-contrast agent injection)	

**Table 2:** Significant radiomic features in Cox regression analysis with risk ratio value based on OS time stratification (short and long OS time).

Group	Feature	Feature Type	Risk Ratio	*p* Value
Immunotherapy group	GLSZM_IBSI_GL_NONUNIF_3D_HU	texture features	21.09	0.02
	GLDZM_IBSI_ZONE_DISTANCE_NONUNIFORMITY_3D_HU		0.00	0.01
	NGLDM_IBSI_GLNONUNIF_2DV_HU		−0.02	0.01
	NGLDM_IBSI_GLNONUNIF_3D_HU		−0.02	0.01
	NGLDM_IBSI_DEP_NONUNIF_2DV_HU		−0.02	0.01
	NGLDM_IBSI_DEP_NONUNIF_3D_HU		−0.06	0.01
	LOG_2D_ENERGY_0_0MM_HU	higher-order statistical features	0.00	0.00

**Table 3:** Significant radiomic features in Cox regression analysis with risk ratio value based on PFS time stratification (short and log PFS time).

Group	Feature	Feature Type	Risk Ratio	*p* Value
Immunotherapy group	GLCM_IBSI_CORRELLATION_2DF_HU	texture features	−3.86	0.01
	GLCM_IBSI_CORRELLATION_2DS_HU		−4.03	0.01
	GLCM_IBSI_CORRELLATION_2DV_HU		−4.06	0.04
	GLCM_IBSI_CORRELLATION_3DF_HU		−4.07	0.00
	GLCM_IBSI_CORRELLATION_3DV_HU		−4.63	0.00
	GLCM_IBSI_FMIC_3DF_HU		−4.88	0.02
	GLCM_IBSI_SMIC_3DV_HU		−4.69	0.02
	GLCM_ASM		349.75	0.00
	GLCM_CORRELATION		−4.65	0.01
	GLCM_CORRELATION_GL		−4.50	0.01
	GLCM_ENERGY		39.37	0.00
	GLCM_ENTROPY		−0.37	0.00
	GLDZM_IBSI_SMALL_DIST_EMPH_2DS_HU		4.00	0.02
	GLDZM_IBSI_ZONE_DISTANCE_ENTROPY_2DS_HU		−0.55	0.01
	GLDZM_IBSI_SMALL_DIST_EMPH_2DV_HU		3.45	0.03
	GLDZM_IBSI_ZONE_DISTANCE_ENTROPY_2DV_HU		−0.47	0.02
	GLDZM_IBSI_ZONE_DISTANCE_NONUNIFORMITY_2DV_HU		4.80	0.03
	GLDZM_IBSI_SMALL_DIST_EMPH_3D_HU		2.84	0.02
	GLDZM_IBSI_SMALL_DIST_LOW_GL_EMPH_3D_HU		119.27	0.00
	GLDZM_IBSI_ZONE_DISTANCE_ENTROPY_3D_HU		−0.45	0.01
	GLSZM_IBSI_ZS_ENTROPY_2DS_HU		−1.09	0.01
	GLSZM_IBSI_ZS_ENTROPY_2DV_HU		−0.98	0.03
	GLSZM_IBSI_ZS_ENTROPY_3D_HU		−0.79	0.04
	NGLDM_IBSI_DEP_ENTROPY_2DF_HU		−0.86	0.02
	NGLDM_IBSI_DEP_ENTROPY_3D_HU		−0.94	0.01
	NGTDM_COARSENESS_2DV_HU		47.72	0.00
	NGTDM_COARSENESS_3D_HU		88.65	0.00
	NGTDM_STRENGTH_2DV_HU		0.13	0.00
	NGTDM_STRENGTH_3D_HU		0.18	0.00
	LOG_2D_ENTROPY_2_5MM_HU	higher-order statistical features	−0.17	0.03
	WAVELET_HHL_PERCENTILE90_HU		−0.01	0.02
	WAVELET_HHL_ENTROPY_HU		−0.17	0.03
Control group	NGLDM_IBSI_GLNONUNIF_2DV_HU	texture features	−2.93	0.01
	NGLDM_IBSI_GLNONUNIF_3D_HU		−0.02	0.00
	NGLDM_IBSI_DEP_NONUNIF_2DV_HU		−0.02	0.00
	NGLDM_IBSI_DEP_NONUNIF_3D_HU		−0.06	0.00
	NGTDM_COMPLEXITY_2DF_HU		0.00	0.04

**Table 4:** Median and range values for textural features significant for stratifying the patients into two groups based on median cutoff of OS time (short and long time). Diagnostic performance is also reported for each significant feature, considering the optimal cutoff value obtained by ROC analysis.

Group	Feature	Feature Type	*p* Value at Kruskal—Wallis Test	Median Value	Minimum Value	Maximum Value
Immunotherapy group	SHIFT_CENTER_OF_MASS_MM	morphological features	0.04	3.75	0.20	63.03
	VOLUME_DENSITY_AEE		0.05	1.17	0.85	1.33
	GLCM_IBSI_CLUSTERPROMINENCE_2DF_HU	texture features	0.03	225,339.31	844.00	2,812,662.20
	GLCM_IBSI_CLUSTERPROMINENCE_2DS_HU		0.03	225,858.72	847.54	2,817,987.50
	GLCM_IBSI_CLUSTERPROMINENCE_2DV_HU		0.03	292,282.78	1011.46	2,989,657.50
	GLCM_IBSI_CLUSTERPROMINENCE_3DF_HU		0.03	214,326.87	778.73	2,815,961.20
	GLCM_IBSI_CLUSTERPROMINENCE_3DV_HU		0.03	220,935.36	783.67	2,819,584.20
	GLDZM_IBSI_GL_VARIANCE_2DS_HU		0.05	82.02	8.15	205.34
	GLDZM_IBSI_GL_VARIANCE_2DV_HU		0.04	99.27	10.18	212.22
	GLDZM_IBSI_GL_VARIANCE_3D_HU		0.03	116.19	19.56	239.15
	GLSZM_IBSI_GL_VARIANCE_2DS_HU		0.05	82.02	8.15	205.34
	GLSZM_IBSI_GL_VARIANCE_2DV_HU		0.04	99.27	10.18	212.22
	GLSZM_IBSI_GL_VARIANCE_3D_HU		0.03	116.19	19.56	239.15
	NGTDM_COMPLEXITY_2DF_HU		0.04	1176.02	79.07	6533.22
	LOG_2D_MEAN_2_5MM_HU	higher-order statistical features	0.04	−0.18	−1.27	2.42
	WAVELET_HHL_ENERGY_HU		0.04	1,998,296.25	43886.58	63,200,000.00
	WAVELET_HHL_MEDIAN_HU		0.05	0.02	−0.39	2.19
	WAVELET_HHL_MIN_HU		0.01	−149.82	−299.53	−68.56
Control group	GREATEST_PLANAR_AXIS	morphological features	0.00	1.00	0.00	2.00
	LOG_2D_MEAN_2_5MM_HU	higher-order statistical features	0.03	−0.18	−1.27	2.42

**Table 5:** Median value and range for textural features significant for stratifying the patients into two groups based on median cutoff of PFS time (short and long time).

Group	Feature	Feature Type	*p* Value at Kruskal—Wallis Test	Median Value	Minimum Value	Maximum Value
Immunotherapy group	ANTPOST_LENGTH_MM	morphological features	0.02	41.52	5.94	111.37
	APPROXIMATE_VOLUME_ML		0.03	26.33	0.23	718.78
	APPROXIMATE_VOLUME_MM3		0.03	26,329.14	231.27	718,778.30
	AVG_AXIAL_DIAMETER_MM		0.01	38.40	6.37	114.50
	AVG_CORONAL_DIAMETER_MM		0.02	36.60	7.46	125.46
	AVG_SAGITTAL_DIAMETER_MM		0.02	41.02	7.42	121.31
	LARGEST_PLANAR_DIAMETER_MM		0.01	47.87	6.78	121.53
	LARGEST_PLANAR_ORTHO_DIAMETER_MM		0.01	29.75	5.97	109.43
	SHIFT_CENTER_OF_MASS_MM		0.03	3.75	0.20	63.03
	COMPACTNESS1_MM		0.04	45.68	3.49	324.34
	CORONAL_LONG_AXIS_MM		0.02	42.04	8.38	139.23
	CRANIALCAUDAL_LENGTH_MM		0.04	37.28	5.67	131.15
	GREATEST_PLANAR_LENGTH		0.02	50.87	8.72	145.10
	SAGITTAL_LONG_AXIS_MM		0.02	50.25	8.72	145.10
	SAGITTAL_SHORT_AXIS_MM		0.04	30.55	3.33	102.60
	SURFACE_AREA_MM2		0.02	5048.57	164.85	44,177.98
	TRANSVERSE_LENGTH_MM		0.02	38.62	6.76	117.99
	VOLUME_ML		0.03	24.48	0.19	718.42
	VOLUME_MM3		0.03	24,480.15	185.97	718,415.94
	VOLUME_VOXELS		0.01	16,348.50	97.00	534,080.00
	VOLUMETRIC_LENGTH_MM		0.01	52.86	8.90	145.30
	L1_DISTANCE_MM		0.02	48.33	7.82	121.30
	L2_DISTANCE_MM		0.03	34.81	6.56	100.44
	L3_DISTANCE_MM		0.03	25.00	4.36	89.54
	SOLID_VOLUME_MM3	lung CT features	0.04	25,469.57	128.75	717,505.20
	SOLID_VOLUME_ML		0.04	25.47	0.13	717.51
	SOLID_VOLUME_VOXELS		0.01	15,791.50	54.00	533,134.00
	PART_SOLID_DIAMETER_MM		0.01	37.89	5.24	114.44
	INTENSITY_HISTOGRAM_ENERGY_HU	features based on intensity value	0.02	37,350,000.00	39,863.00	1,450,000,000.00
	GLCM_IBSI_CORRELLATION_2DF_HU	texture features	0.00	0.72	0.33	0.89
	GLCM_IBSI_CORRELLATION_2DS_HU		0.00	0.72	0.34	0.89
	GLCM_IBSI_CORRELLATION_2DV_HU		0.03	0.80	0.42	0.94
	GLCM_IBSI_CORRELLATION_3DF_HU		0.01	0.58	0.08	0.89
	GLCM_IBSI_CORRELLATION_3DV_HU		0.01	0.59	0.22	0.89
	GLCM_IBSI_FMIC_3DV_HU		0.00	−0.11	−0.25	−0.03
	GLCM_IBSI_SMIC_3DV_HU		0.01	0.74	0.46	0.92
	GLCM_ASM		0.01	0.00	0.00	0.01
	GLCM_CORRELATION		0.01	0.64	0.39	0.91
	GLCM_CORRELATION_GL		0.01	0.64	0.00	0.90
	GLCM_ENERGY		0.01	0.01	0.01	0.10
	GLCM_ENTROPY		0.00	12.89	6.60	14.86
	GLDZM_IBSI_SMALL_DIST_EMPH_2DS_HU		0.02	0.41	0.18	0.67
	GLDZM_IBSI_ZONE_DISTANCE_ENTROPY_2DS_HU		0.00	6.43	4.28	8.09
	GLDZM_IBSI_ZONE_DISTANCE_NONUNIFORMITY_2DS_HU		0.00	45.38	11.58	90.68
	GLDZM_IBSI_ZONE_DISTANCE_NONUNIFORMITY_NORMALIZED_2DS_HU		0.03	0.22	0.07	0.45
	GLDZM_IBSI_ZONE_DISTANCE_VARIANCE_2DS_HU		0.05	8.07	0.52	149.19
	GLDZM_IBSI_GL_NONUNIFORMITY_2DV_HU		0.02	255.74	3.26	9626.30
	GLDZM_IBSI_SMALL_DIST_EMPH_2DV_HU		0.02	0.35	0.13	0.63
	GLDZM_IBSI_ZONE_DISTANCE_ENTROPY_2DV_HU		0.01	7.68	5.26	9.16
	GLDZM_IBSI_ZONE_DISTANCE_NONUNIFORMITY_2DV_HU		0.00	777.59	33.98	7437.29
	GLDZM_IBSI_ZONE_DISTANCE_NONUNIFORMITY_NORMALIZED_2DV_HU		0.03	0.16	0.04	0.40
	GLDZM_IBSI_GL_NONUNIFORMITY_3D_HU		0.00	109.21	2.64	1697.68
	GLDZM_IBSI_LARGE_DIST_EMPH_3D_HU		0.01	5.64	1.00	93.17
	GLDZM_IBSI_SMALL_DIST_EMPH_3D_HU		0.01	0.68	0.35	1.00
	GLDZM_IBSI_SMALL_DIST_LOW_GL_EMPH_3D_HU		0.02	0.00	0.00	0.03
	GLDZM_IBSI_ZONE_DISTANCE_ENTROPY_3D_HU		0.03	6.67	4.40	8.57
	GLDZM_IBSI_ZONE_DISTANCE_NONUNIFORMITY_3D_HU		0.00	1097.70	52.00	6943.99
	GLDZM_IBSI_ZONE_DISTANCE_NONUNIFORMITY_NORMALIZED_3D_HU		0.01	0.43	0.13	1.00
	GLDZM_IBSI_ZONE_DISTANCE_VARIANCE_3D_HU		0.01	1.76	0.00	53.91
	GLRLM_IBSI_GLNONUNIFORMITY_2DV_HU		0.04	4350.77	14.15	254,334.25
	GLRLM_IBSI_GLNONUNIFORMITY_3DF_HU		0.03	1171.83	3.58	69,500.89
	GLRLM_IBSI_GLNONUNIFORMITY_3DV_HU		0.03	15,231.78	46.31	903,435.10
	GLRLM_IBSI_RUNLENGTHNONUNIFORMITY_2DF_HU		0.01	374.04	29.69	2766.90
	GLRLM_IBSI_RUNLENGTHNONUNIFORMITY_2DS_HU		0.01	1491.65	118.62	11,004.87
	GLRLM_IBSI_RUNLENGTHNONUNIFORMITY_2DV_HU		0.01	29,137.73	355.64	612,642.30
	GLRLM_IBSI_RUNLENGTHNONUNIFORMITY_3DF_HU		0.01	9072.57	91.34	177,585.50
	GLRLM_IBSI_RUNLENGTHNONUNIFORMITY_3DV_HU		0.01	117,863.44	1187.01	2,296,799.20
	GLSZM_IBSI_GL_NONUNIF_2DV_HU		0.02	255.74	3.26	9626.30
	GLSZM_IBSI_GL_NONUNIF_3D_HU		0.00	109.21	2.64	1697.68
	GLSZM_IBSI_SMALL_ZONE_EMPH_3D_HU		0.03	0.74	0.60	0.83
	GLSZM_IBSI_SMALL_ZONE_LOW_GL_EMPH_3D_HU		0.04	0.00	0.00	0.03
	GLSZM_IBSI_ZS_ENTROPY_2DS_HU		0.00	5.70	4.24	6.37
	GLSZM_IBSI_ZS_ENTROPY_2DV_HU		0.03	6.52	5.16	7.07
	GLSZM_IBSI_ZS_ENTROPY_3D_HU		0.03	6.62	5.32	7.44
	GLSZM_IBSI_ZS_NONUNIF_NORMALISED_3D_HU		0.03	0.51	0.33	0.65
	GLSZM_IBSI_ZS_NONUNIF_2DS_HU		0.00	130.66	23.54	725.63
	GLSZM_IBSI_ZS_NONUNIF_2DV_HU		0.00	2406.05	69.56	34,717.15
	GLSZM_IBSI_ZS_NONUNIF_3D_HU		0.00	1367.97	44.19	13,020.40
	NGLDM_IBSI_GLNONUNIF_2DV_HU		0.04	2108.79	3.66	118,346.81
	NGLDM_IBSI_GLNONUNIF_3D_HU		0.04	2108.79	3.66	118,346.81
	NGLDM_IBSI_DEP_ENTROPY_2DF_HU		0.01	5.55	4.28	6.48
	NGLDM_IBSI_DEP_ENTROPY_3D_HU		0.01	7.00	5.52	7.89
	NGLDM_IBSI_DEP_NONUNIF_2DF_HU		0.01	185.29	22.44	1201.75
	NGLDM_IBSI_DEP_NONUNIF_2DV_HU		0.01	3029.10	64.51	82,117.83
	NGLDM_IBSI_DEP_NONUNIF_3D_HU		0.00	1532.28	43.76	32,843.65
	NGLDM_IBSI_HIGH_DEP_LOW_GL_EMPH_3D_HU		0.04	0.03	0.01	0.21
	NGTDM_BUSYNESS_2DV_HU		0.02	0.43	0.03	9.34
	NGTDM_BUSYNESS_3D_HU		0.01	0.58	0.06	11.04
	NGTDM_COARSENESS_2DV_HU		0.03	0.00	0.00	0.05
	NGTDM_COARSENESS_3D_HU		0.01	0.00	0.00	0.04
	NGTDM_STRENGTH_2DV_HU		0.00	3.35	0.24	33.97
	NGTDM_STRENGTH_3D_HU		0.00	2.01	0.19	23.53
	LOG_2D_ENERGY_0_0MM_HU	higher-order statistical features	0.00	628,000,000.00	0.00	15,700,000,000.00
	LOG_2D_ENERGY_2_5MM_HU		0.00	462,665.47	5610.69	10,400,000.00
	LOG_2D_ENTROPY_2_5MM_HU		0.01	14.00	6.60	19.03
	LOG_2D_MEAN_2_5MM_HU		0.01	−0.18	−1.27	2.42
	WAVELET_HHL_ENERGY_HU		0.01	1,998,296.25	43,886.58	63,200,000.00
	WAVELET_HHL_ENTROPY_HU		0.01	14.00	6.60	19.03
	WAVELET_HHL_KURTOSIS_HU		0.04	29.91	2.04	324.52
	WAVELET_HHL_PERCENTILE10_HU		0.03	−7.04	−35.16	−2.24
	WAVELET_HHL_PERCENTILE90_HU		0.04	7.53	2.31	33.86
	WAVELET_HHL_ROBUST_MEAN_DEVIATION_HU		0.05	2.53	0.93	13.78
Control group	SHIFT_CENTER_OF_MASS_MM	morphological features	0.03	3.75	0.20	63.03
	PERCENT_AIR	lung CT features	0.02	0.00	0.00	1.05
	NGLDM_IBSI_DEP_VARIANCE_2DF_HU	texture features	0.03	2.57	0.19	5.69
	LOG_2D_COV_2_5MM_HU	higher-order statistical features	0.01	−9.83	−1429.77	308.28

## Data Availability

Data are contained within the article.

## Abbreviations

ACC	accuracy
ANN	artificial neural network
AUC	area under ROC curve
CT	computed tomography
CDSS	clinical decision support systems
DT	decision tree
KNN	*k*-nearest neighbor
GLCM	grey-level co-occurrence matrix
GLDZM	grey-level distance zone matrix
GLRLM	grey-level run length matrix
GLSZM	grey-level size zone matrix
LASSO	least absolute shrinkage and selection operator
LDA	linear discrimination analysis
LOG	Laplacian of Gaussian
NSCLC	non-small cell lung cancer
NGLDM	neighboring grey-level dependence matrix
NGTDM	neighboring grey-tone difference matrix
NPV	negative predictive value
OS	overall survival
PFS	progression-free survival
PPV	positive predictive value
QIDS	quantitative imaging decision support
SENS	sensitivity
SPEC	specificity
SVM	support vector machine
ROC	receiver operating characteristic
RPM	rapid precise metrics
WHO	World Health Organization

## Appendix A

**Metric Code**	**Name**	**Feature Type**	**Units**	**IBSI Compliance**	**Description**
SOLID_VOLUME_ML	Solid Density Volume	lung CT features	mL		Volume of the solid density of the specified ROI in milliliters.
SOLID_VOLUME_MM3	Solid Density Volume		mm^3		Volume of the solid density of the specified ROI in cubic millimeters.
SOLID_VOLUME_VOXELS	Solid Density Volume		voxels		Volume of the solid density of the specified ROI in voxels.
PART_SOLID_DIAMETER_MM	Part-Solid Diameter		mm		The average diameter of the solid portions of a part-solid lesion.
PERCENT_AIR	Percent Air				The estimated percent of volume that is air in this ROI.
ANTPOST_LENGTH_MM	Anterior-Posterior Length	morphological features	mm		A measure of the anterior-posterior distance.
APPROXIMATE_VOLUME_ML	Approximate Volume		mL		Approximate volume of the specified ROI of the image in milliliters. For studies with gantry tilt, PARALLELEPIPED_VOLUME_MM3 is recommended.
APPROXIMATE_VOLUME_MM3	Approximate Volume		mm^3		Approximate volume of the specified ROI of the image in cubic millimeters assuming water equivalent. For studies with gantry tilt, PARALLELEPIPED_VOLUME_MM3 is recommended.
AVG_AXIAL_DIAMETER_MM	Average Axial Diameter		mm		The average of largest axial planar and orthogonal diameters, in millimeters
AVG_CORONAL_DIAMETER_MM	Average Coronal Diameter		mm		The average of largest coronal planar and orthogonal diameters, in millimeters
AVG_SAGITTAL_DIAMETER_MM	Average Sagittal Diameter		mm		The average of largest sagittal planar and orthogonal diameters, in millimeters
COMPACTNESS1_MM	Mesh Compactness 1		mm^5/3	1	IBSI-consistent dimensionful measure of compactness of ROI, independent of scale and orientation (first of three implementations), using standard unit shape-derived information.
CORONAL_LONG_AXIS_MM	Coronal Long Axis		mm		A measure of the longest straight line that can fit entirely inside an XZ-planar slice of the 3D structure (from edge to edge, without ever leaving structure), in millimeters.
CRANIALCAUDAL_LENGTH_MM	Cranial-Caudal Length		mm		A measure of the cranial-caudal distance.
VOLUME_DENSITY_AEE	Volume Density-Approximate Enclosing Ellipsoid			1	IBSI-consistent volume fraction of the approximate enclosing ellipsoid occupied by the ROI
VOLUME_ML	Volume		mL		IBSI-consistent volume of the specified ROI of the image in milliliters.
VOLUME_MM3	Volume		mm^3		IBSI-consistent volume of the specified ROI of the image in cubic millimeters.
VOLUME_VOXELS	Volume		voxels		IBSI-consistent approximate volume derived from voxel count inside ROI
VOLUMETRIC_LENGTH_MM	Volumetric Length		mm		A measure of the longest straight line that can fit entirely inside the 3D structure (from edge to edge, without ever leaving structure).
GREATEST_PLANAR_AXIS	Greatest Planar Axis Length		mm		
GREATEST_PLANAR_LENGTH	Greatest Planar Long Axis Length		mm		Greatest length among the sagittal longest axis, axial longest axis, and coronal longest axis lengths.
L1_DISTANCE_MM	Long (L1) Full Axis Length		mm		IBSI-consistent length of the long (L1) full principal axis, in millimeters, from edge to edge of the ROI.
L2_DISTANCE_MM	Short (L2) Full Axis Length		mm		IBSI-consistent length of the short (L2) full principal axis, in millimeters, from edge to edge of the ROI.
L3_DISTANCE_MM	Normal (L3) Full Axis Length		mm		IBSI-consistent length of the normal (L3) full principal axis, in millimeters, from edge to edge of the ROI.
LARGEST_PLANAR_DIAMETER_MM	Axial Long Axis		mm		A measure of the longest straight line that can fit entirely inside an XY-planar slice of the 3D structure (from edge to edge, without ever leaving structure), in millimeters.
LARGEST_PLANAR_ORTHO_DIAMETER_MM	Axial Short Axis		mm		A measure of the longest orthogonal line to the longest planar line, that can fit entirely inside an XY-planar slice of the 3D structure (from edge to edge, without ever leaving structure), in millimeters
SAGITTAL_LONG_AXIS_MM	Sagittal Long Axis		mm		A measure of the longest straight line that can fit entirely inside an YZ-planar slice of the 3D structure (from edge to edge, without ever leaving structure), in millimeters.
SAGITTAL_SHORT_AXIS_MM	Sagittal Short Axis		mm		A measure of the longest orthogonal line to the longest planar line, that can fit entirely inside an YZ-planar slice of the 3D structure (from edge to edge, without ever leaving structure), in millimeters
SHIFT_CENTER_OF_MASS_MM	Center of Mass Shift		mm	1	IBSI-consistent shift in the center of mass due to image intensity.
SURFACE_AREA_MM2	Surface Area		mm^2	1	IBSI-consistent surface area of the specified ROI of the image in square millimeters.
TRANSVERSE_LENGTH_MM	Transverse Length		mm		A measure of the transverse distance.
INTENSITY_HISTOGRAM_ENERGY_HU	Intensity Histogram Energy	Intensity-based feature		1	IBSI-consistent intensity histogram energy of all voxels in ROI binned for PET.
GLCM_ASM	GLCM Avg Angular Second Moment	textural features			Average angular second moments of GLCM in all 26 directions. Raw HU used, unbinned and with background padding 1 voxel around the ROI.
GLCM_CORRELATION	GLCM Avg Correlation				Average correlations of GLCM in all 26 directions. Raw HU used, unbinned and with background padding 1 voxel around the ROI.
GLCM_CORRELATION_GL	GLCM Avg Correlation for Grey Leveled Image				Average correlations of GLCM in all 26 directions for grey leveled CT or PET image with background padding 1 voxel around the ROI.
GLCM_ENERGY	GLCM Avg Energy				Average energies of GLCM in all 26 directions. Raw HU used, unbinned and with background padding 1 voxel around the ROI.
GLCM_ENTROPY	GLCM Avg Entropy				Average entropies of GLCM in all 26 directions. Raw HU used, unbinned and with background padding 1 voxel around the ROI.
GLCM_IBSI_CLUSTERPROMINENCE_2DF_HU	GLCM Cluster Prominence for Grey Leveled Image from IBSI by Slice without Merging			1	IBSI-consistent cluster prominence of GLCM of unpadded ROI binned for CT with aggregation by slice without merging.
GLCM_IBSI_CLUSTERPROMINENCE_2DS_HU	GLCM Cluster Prominence for Grey Leveled Image from IBSI by Slice with Merging by Slice			1	IBSI-consistent cluster prominence of GLCM of unpadded ROI binned for CT with aggregation by slice with merging by slice.
GLCM_IBSI_CLUSTERPROMINENCE_2DV_HU	GLCM Cluster Prominence for Grey Leveled Image from IBSI by Slice with Merging			1	IBSI-consistent cluster prominence of GLCM of unpadded ROI binned for CT with aggregation by slice with merging.
GLCM_IBSI_CLUSTERPROMINENCE_3DF_HU	GLCM Cluster Prominence for Grey Leveled Image from IBSI by Volume without Merging			1	IBSI-consistent cluster prominence of GLCM of unpadded ROI binned for CT with aggregation by volume without merging. binning
GLCM_IBSI_CLUSTERPROMINENCE_3DV_HU	GLCM Cluster Prominence for Grey Leveled Image from IBSI by Volume with Full Merging			1	IBSI-consistent cluster prominence of GLCM of unpadded ROI binned for CT with aggregation by volume with full merging. binning
GLCM_IBSI_CORRELLATION_2DF_HU	GLCM Correlation for the Grey Leveled Image from the IBSI by Slice without Merging			1	IBSI-consistent correlation of GLCM ROI binned for CT with aggregation by slice without merging.
GLCM_IBSI_CORRELLATION_2DS_HU	GLCM Correlation for the Grey Leveled Image from the IBSI by Slice with Merging by Slice			1	IBSI-consistent correlation of GLCM ROI binned for CT with aggregation by slice with merging by slice.
GLCM_IBSI_CORRELLATION_2DV_HU	GLCM Correlation for the Grey Leveled Image from the IBSI by Slice with Merging			1	IBSI-consistent correlation of GLCM ROI binned for CT with aggregation by slice with merging.
GLCM_IBSI_CORRELLATION_3DF_HU	GLCM Correlation for the Grey Leveled Image from the IBSI by Volume without Merging			1	IBSI-consistent correlation of GLCM ROI binned for CT with aggregation by volume without merging.
GLCM_IBSI_CORRELLATION_3DV_HU	GLCM Correlation for the Grey Leveled Image from the IBSI by Volume with Full Merging			1	IBSI-consistent correlation of GLCM ROI binned for CT with aggregation by volume with full merging.
GLCM_IBSI_FMIC_3DV_HU	GLCM First Measure of InformationCorrelation for Grey Leveled Image from IBSI by Volume with Full Merging			1	IBSI-consistent first measure of information correlation of GLCM of unpadded ROI binned for CT with aggregation by volume with full merging.
GLCM_IBSI_SMIC_3DV_HU	GLCM Second Measure of Information Correlation for Grey Leveled Image from IBSI by Slice with Full Merging			1	IBSI-consistent second measure of inform of unpadded ROI binned for CT with aggregation by slice with full merging.
GLDZM_IBSI_GL_NONUNIFORMITY_2DV_HU	GLDZM Grey Level Nonuniformity with Merging by Slice from CT			1	IBSI-consistent grey level nonuniformity of GLDZM of unpadded ROI binned for CT from 8 directions in 2 dimensions with merging.
GLDZM_IBSI_GL_NONUNIFORMITY_3D_HU	GLDZM Grey Level Nonuniformity from CT			1	IBSI-consistent grey level nonuniformity of GLDZM of unpadded ROI binned for CT from 26 directions in 3 dimensions.
GLDZM_IBSI_GL_VARIANCE_2DS_HU	GLDZM Grey Level Variance from CT without Merging			1	IBSI-consistent grey level variance of GLDZM of unpadded ROI binned for CT from 8 directions in 2 dimensions.
GLDZM_IBSI_GL_VARIANCE_2DV_HU	GLDZM Grey Level Variance with Merging by Slice from CT			1	IBSI-consistent grey level variance of GLDZM of unpadded ROI binned for CT from 8 directions in 2 dimensions with merging.
GLDZM_IBSI_GL_VARIANCE_3D_HU	GLDZM Grey Level Variance from CT			1	IBSI-consistent grey level variance of GLDZM of unpadded ROI binned for CT from 26 directions in 3 dimensions.
GLDZM_IBSI_LARGE_DIST_EMPH_3D_HU	GLDZM Large Distance Emphasis with Full Merging from CT			1	IBSI-consistent large distance emphasis of GLDZM of unpadded ROI binned for CT from 26 directions in 3 dimensions.
GLDZM_IBSI_SMALL_DIST_EMPH_2DS_HU	GLDZM Small Distance Emphasis without Merging from CT			1	IBSI-consistent small distance emphasis of GLDZM of unpadded ROI binned for CT from 8 directions in 2 dimensions.
GLDZM_IBSI_SMALL_DIST_EMPH_2DV_HU	GLDZM Small Distance Emphasis with Merging by Slice from CT			1	IBSI-consistent small distance emphasis of GLDZM of unpadded ROI binned for CT from 8 directions in 2 dimensions with merging.
GLDZM_IBSI_SMALL_DIST_EMPH_3D_HU	GLDZM Small Distance Emphasis with Full Merging from CT			1	IBSI-consistent small distance emphasis of GLDZM of unpadded ROI binned for CT from 26 directions in 3 dimensions.
GLDZM_IBSI_SMALL_DIST_LOW_GL_EMPH_3D_HU	GLDZM Small Distance Low Grey Level Emphasis with Full Merging from CT			1	IBSI-consistent small distance low grey level emphasis of GLDZM of unpadded ROI binned for CT from 26 directions in 3 dimensions.
GLDZM_IBSI_ZONE_DISTANCE_ENTROPY_2DS_HU	GLDZM Zone Distance Entropy without Merging from CT			1	IBSI-consistent grey level nonuniformity of GLDZM of unpadded ROI binned for CT from 8 directions in 2 dimensions.
GLDZM_IBSI_ZONE_DISTANCE_ENTROPY_2DV_HU	GLDZM Zone Distance Entropy with Mergning by Slice from CT			1	IBSI-consistent grey level nonuniformity of GLDZM of unpadded ROI binned for CT from 8 directions in 2 dimensions with merging.
GLDZM_IBSI_ZONE_DISTANCE_ENTROPY_3D_HU	GLDZM Zone Distance Entropy from CT			1	IBSI-consistent grey level nonuniformity of GLDZM of unpadded ROI binned for CT from 26 directions in 3 dimensions.
GLDZM_IBSI_ZONE_DISTANCE_NONUNIFORMITY_2DS_HU	GLDZM Zone Distance Nonuniformity without Merging from CT			1	IBSI-consistent grey level nonuniformity of GLDZM of unpadded ROI binned for CT from 8 directions in 2 dimensions.
GLDZM_IBSI_ZONE_DISTANCE_NONUNIFORMITY_2DV_HU	GLDZM Zone Distance Nonuniformity with Merging by Slice from CT			1	IBSI-consistent grey level nonuniformity of GLDZM of unpadded ROI binned for CT from 8 directions in 2 dimensions with merging.
GLDZM_IBSI_ZONE_DISTANCE_NONUNIFORMITY_3D_HU	GLDZM Zone Distance Nonuniformity from CT			1	IBSI-consistent grey level nonuniformity of GLDZM of unpadded ROI binned for CT from 26 directions in 3 dimensions.
GLDZM_IBSI_ZONE_DISTANCE_NONUNIFORMITY_NORMALIZED_2DS_HU	GLDZM Zone Distance Nonuniformity Normalised without Merging from CT			1	IBSI-consistent grey level normalized nonuniformity of GLDZM of unpadded ROI binned for CT from 8 directions in 2 dimensions.
GLDZM_IBSI_ZONE_DISTANCE_NONUNIFORMITY_NORMALIZED_2DV_HU	GLDZM Zone Distance Nonuniformity Normalised with Mergning by Slice from CT			1	IBSI-consistent grey level normalized nonuniformity of GLDZM of unpadded ROI binned for CT from 8 directions in 2 with merging dimensions.
GLDZM_IBSI_ZONE_DISTANCE_NONUNIFORMITY_NORMALIZED_3D_HU	GLDZM Zone Distance Nonuniformity Normalised from CT			1	IBSI-consistent grey level normalized nonuniformity of GLDZM of unpadded ROI binned for CT from 26 directions in 3 dimensions.
GLDZM_IBSI_ZONE_DISTANCE_VARIANCE_2DS_HU	GLDZM Zone Distance Variance without Merging from CT			1	IBSI-consistent grey level variance of GLDZM of unpadded ROI binned for CT from 8 directions in 2 dimensions.
GLDZM_IBSI_ZONE_DISTANCE_VARIANCE_3D_HU	GLDZM Zone Distance Variance from CT			1	IBSI-consistent grey level variance of GLDZM of unpadded ROI binned for CT from 26 directions in 3 dimensions.
GLRLM_IBSI_GLNONUNIFORMITY_2DV_HU	GLRLM Grey Level Nonuniformity by Slice with Full Merging from CT			1	IBSI-consistent grey levelnonuniformity from GLRLM of unpadded ROI binned for CT from merging in 8 directions for each slice.
GLRLM_IBSI_GLNONUNIFORMITY_3DF_HU	GLRLM Grey Level Nonuniformityas Volume, without Merging from CT			1	IBSI-consistent grey levelnonuniformity from GLRLM of unpadded ROI binned for CT from averaging 26 directions in 3 dimensions.
GLRLM_IBSI_GLNONUNIFORMITY_3DV_HU	GLRLM Grey Level Nonuniformity as Volume, with Full Merging from CT			1	IBSI-consistent grey levelnonuniformity from GLRLM of unpadded ROI binned for CT from merging 26 directions in 3 dimensions.
GLRLM_IBSI_RUNLENGTHNONUNIFORMITY_2DF_HU	GLRLM Run Length Nonuniformity by Slice without Merging from CT			1	IBSI-consistent run length nonuniformity from GLRLM of unpadded ROI binned for CT from averaging in 8 directions for each slice.
GLRLM_IBSI_RUNLENGTHNONUNIFORMITY_2DS_HU	GLRLM Run Length Nonuniformity by Slice with Merging by Slice from CT			1	IBSI-consistent run length nonuniformity from GLRLM of unpadded ROI binned for CT from merging matrices from each slice and averaging the result.
GLRLM_IBSI_RUNLENGTHNONUNIFORMITY_2DV_HU	GLRLM Run Length Nonuniformity by Slice with Full Merging from CT			1	IBSI-consistent run length nonuniformity from GLRLM of unpadded ROI binned for CT from merging in 8 directions for each slice.
GLRLM_IBSI_RUNLENGTHNONUNIFORMITY_3DF_HU	GLRLM Run Length Nonuniformity as Volume, without Merging from CT			1	IBSI-consistent run length nonuniformity from GLRLM of unpadded ROI binned for CT from averaging 26 directions in 3 dimensions.
GLRLM_IBSI_RUNLENGTHNONUNIFORMITY_3DV_HU	GLRLM Run Length Nonuniformity as Volume, with Full Merging from CT			1	IBSI-consistent run length nonuniformity from GLRLM of unpadded ROI binned for CT from merging 26 directions in 3 dimensions.
GLSZM_IBSI_GL_NONUNIF_2DV_HU	GLSZM Grey Level Nonuniformity by Slice, with Full Merging			1	IBSI-consistent grey level nonuniformity of GLSZM of unpadded ROI binned for CT with aggregation by 2D volume.
GLSZM_IBSI_GL_NONUNIF_3D_HU	GLSZM Grey Level Nonuniformity as Volume, with Full Merging			1	IBSI-consistent grey level nonuniformity of GLSZM of unpadded ROI binned for CT with aggregation by 3D volume.
GLSZM_IBSI_GL_VARIANCE_2DS_HU	GLSZM Grey Level Variance by Slice, with Merging by Slice			1	IBSI-consistent grey level variance of GLSZM of unpadded ROI binned for CT with aggregation by slice.
GLSZM_IBSI_GL_VARIANCE_2DV_HU	GLSZM Grey Level Variance by Slice, with Full Merging			1	IBSI-consistent grey level variance of GLSZM of unpadded ROI binned for CT with aggregation by 2D volume.
GLSZM_IBSI_GL_VARIANCE_3D_HU	GLSZM Grey Level Variance as Volume, with Full Merging			1	IBSI-consistent grey level variance of GLSZM of unpadded ROI binned for CT with aggregation by 3D volume.
GLSZM_IBSI_SMALL_ZONE_EMPH_3D_HU	GLSZM Small Zone Emphasis as Volume, with Full Merging			1	IBSI-consistent small zone emphasis of GLSZM of unpadded ROI binned for CT with aggregation by 3D volume.
GLSZM_IBSI_SMALL_ZONE_LOW_GL_EMPH_3D_HU	GLSZM Small Zone Low Grey Level Emphasis as Volume, with Full Merging			1	IBSI-consistent small zone low grey level emphasis of GLSZM of unpadded ROI binned for CT with aggregation by 3D volume.
GLSZM_IBSI_ZS_ENTROPY_2DS_HU	GLSZM Zone Size Entropy by Slice, with Merging by Slice			1	IBSI-consistent zone size entropy from GLSZM of unpadded ROI binned for CT with aggregation by slice.
GLSZM_IBSI_ZS_ENTROPY_2DV_HU	GLSZM Zone Size Entropy by Slice, with Full Merging			1	IBSI-consistent zone size entropy from GLSZM of unpadded ROI binned for CT with aggregation by 2D volume.
GLSZM_IBSI_ZS_ENTROPY_3D_HU	GLSZM Zone Size Entropy as Volume, with Full Merging			1	IBSI-consistent zone size entropy from GLSZM of unpadded ROI binned for CT with aggregation by 3D volume.
GLSZM_IBSI_ZS_NONUNIF_2DS_HU	GLSZM Zone Size Nonuniformity by Slice, with Merging by Slice			1	IBSI-consistent zone size uniformity from GLSZM of unpadded ROI binned for CT with aggregation by slice.
GLSZM_IBSI_ZS_NONUNIF_2DV_HU	GLSZM Zone Size Nonuniformity by Slice, with Full Merging			1	IBSI-consistent zone size uniformity from GLSZM of unpadded ROI binned for CT with aggregation by 2D volume.
GLSZM_IBSI_ZS_NONUNIF_3D_HU	GLSZM Zone Size Nonuniformity as Volume, with Full Merging			1	IBSI-consistent zone size uniformity from GLSZM of unpadded ROI binned for CT with aggregation by 3D volume.
GLSZM_IBSI_ZS_NONUNIF_NORMALISED_3D_HU	GLSZM Normalised Zone Size Nonuniformity as Volume, with Full Merging			1	IBSI-consistent normalizedzone size nonuniformity of GLSZM of unpadded ROI binned for CT with aggregation by 3D volume.
NGLDM_IBSI_DEP_ENTROPY_2DF_HU	NGLDM Dependence Entropy by Slice, without Merging			1	IBSI-consistent dependence entropy of NGLDM of unpadded ROI binned for CT with aggregation by slice without merging.
NGLDM_IBSI_DEP_ENTROPY_3D_HU	NGLDM Dependence Entropy as Volume, with Full Merging			1	IBSI-consistent dependence entropy of NGLDM of unpadded ROI binned for CT with aggregation by volume with full merging.
NGLDM_IBSI_DEP_NONUNIF_2DF_HU	NGLDM Dependence Nonuniformity by Slice, without Merging			1	IBSI-consistent dependence nonuniformity of NGLDM of unpadded ROI binned for CT with aggregation by slice without merging.
NGLDM_IBSI_DEP_NONUNIF_2DV_HU	NGLDM Dependence Nonuniformity by Slice, with Merging by Slice			1	IBSI-consistent dependence nonuniformity of NGLDM of unpadded ROI binned for CT with aggregation by slice with merging.
NGLDM_IBSI_DEP_NONUNIF_3D_HU	NGLDM Dependence Nonuniformity as Volume, with Full Merging			1	IBSI-consistent dependence nonuniformity of NGLDM of unpadded ROI binned for CT with aggregation by volume with full merging.
NGLDM_IBSI_DEP_VARIANCE_2DF_HU	NGLDM Dependence Variance by Slice, without Merging			1	IBSI-consistent dependence entropy of NGLDM of unpadded ROI binned for CT with aggregation by slice without merging.
NGLDM_IBSI_GLNONUNIF_2DV_HU	NGLDM GL Nonuniformity by Slice, with Merging by Slice			1	IBSI-consistent grey level nonuniformity of NGLDM of unpadded ROI binned for CT with aggregation by slice with merging.
NGLDM_IBSI_GLNONUNIF_3D_HU	NGLDM GL Nonuniformity as Volume, with Full Merging			1	IBSI-consistent grey level nonuniformity of NGLDM of unpadded ROI binned for CT with aggregation by volume with full merging.
NGLDM_IBSI_HIGH_DEP_LOW_GL_EMPH_3D_HU	NGLDM High Dependence Low GL Emphasis as Volume, with Full Merging			1	IBSI-consistent high dependence low grey level of NGLDM of unpadded ROI binned for CT with aggregation by volume with full merging.
NGTDM_BUSYNESS_2DV_HU	NGTDM Busyness by Slice with Full Merging			1	IBSI-consistent busyness of NGTDM of unpadded ROI with aggregation by merging 8 matrices for each slice
NGTDM_BUSYNESS_3D_HU	NGTDM Busyness as Volume, with Full Merging			1	IBSI-consistent busyness of NGTDM of unpadded ROI with aggregation by merging matrices from all 26 directions
NGTDM_COARSENESS_2DV_HU	NGTDM Coarseness, by Slice with Full Merging			1	IBSI-consistent coarseness of NGTDM of unpadded ROI with aggregation by merging 8 matrices for each slice
NGTDM_COARSENESS_3D_HU	NGTDM Coarseness, as Volume, with Full Merging			1	IBSI-consistent coarseness of NGTDM of unpadded ROI with aggregation by merging matrices from all 26 directions
NGTDM_COMPLEXITY_2DF_HU	NGTDM Complexity by Slice without Merging			1	IBSI-consistent complexity of NGTDM of unpadded ROI with aggregation by averaging metrics from all matrices
NGTDM_STRENGTH_2DV_HU	NGTDM Strength by Slice with Full Merging			1	IBSI-consistent strength of NGTDM of unpadded ROI with aggregation by merging 8 matrices for each slice
NGTDM_STRENGTH_3D_HU	NGTDM Strength as Volume, with Full Merging			1	IBSI-consistent strength of NGTDM of unpadded ROI with aggregation by merging matrices from all 26 directions
LOG_2D_COV_2_5MM_HU	Coefficient of Variation of LoG (2.5 mm) Filtered Slice by Slice	higher order statistics features			Coefficient of variation of 2D LoG transformed voxels at 2.5 mm smoothing
LOG_2D_ENERGY_0_0MM_HU	Energy of LoG (0.0 mm) Filtered Slice by Slice		HU^2		Energy of 2D LoG transformed voxels at 0 mm smoothing
LOG_2D_ENERGY_2_5MM_HU	Energy of LoG (2.5 mm) Filtered Slice by Slice		HU^2		Energy of 2D LoG transformed voxels at 2.5 mm smoothing
LOG_2D_ENTROPY_2_5MM_HU	Entropy of LoG (2.5 mm) Filtered Slice by Slice				Entropy of 2D LoG transformed voxels at 2.5 mm smoothing
LOG_2D_MEAN_2_5MM_HU	Mean of LoG (2.5 mm) Filtered Slice by Slice		HU		Mean of 2D LoG transformed voxels at 2.5 mm smoothing
WAVELET_HHL_ENERGY_HU	Wavelet HHL Energy		HU^2		Energy of voxels under wavelet transforms with filters HHL.
WAVELET_HHL_ENTROPY_HU	Wavelet HHL Entropy				Entropy of voxels under wavelet transforms with filters HHL.
WAVELET_HHL_KURTOSIS_HU	Wavelet HHL Excess Kurtosis				Excess kurtosis voxels under wavelet transforms with filters HHL.
WAVELET_HHL_MEDIAN_HU	Wavelet HHL Median		HU		Median of voxels under wavelet transforms with filters HHL.
WAVELET_HHL_MIN_HU	Wavelet HHL MInimum		HU		Minimum of voxels under wavelet transforms with filters HHL.
WAVELET_HHL_PERCENTILE10_HU	Wavelet HHL 10th Percentile		HU		The 10th percentile of voxels under wavelet transforms with filters HHL.
WAVELET_HHL_PERCENTILE90_HU	Wavelet HHL 90th Percentile		HU		The 90th percentile voxels under wavelet transforms with filters HHL.
WAVELET_HHL_ROBUST_MEAN_DEVIATION_HU	Wavelet HHL Robust Mean Deviation		HU		Robust absolute deviation from the mean of voxels under wavelet transforms with filters HHL.
